# Spi-B alleviates food allergy by securing mucosal barrier and immune tolerance in the intestine

**DOI:** 10.3389/falgy.2022.996657

**Published:** 2022-10-06

**Authors:** Narumi Ishihara, Yutaka Nakamura, Kyosuke Yakabe, Seiga Komiyama, Yumiko Fujimura, Tsuneyasu Kaisho, Shunsuke Kimura, Koji Hase

**Affiliations:** ^1^ Division of Biochemistry, Faculty of Pharmacy, School of Pharmaceutical Sciences, Keio University, Tokyo, Japan; ^2^ Department of Microbiology and Immunology, School of Pharmaceutical Sciences, Wakayama Medical University, Wakayama, Japan; ^3^ Department of Immunology, Institute of Advanced Medicine, Wakayama Medical University, Wakayama, Japan; ^4^ Laboratory for Inflammatory Regulation, RIKEN Center for Integrative Medical Science (IMS-RCAI), Yokohama, Japan; ^5^ Precursory Research for Embryonic Science and Technology (PRESTO), Japan Science and Technology Agency, Saitama, Japan; ^6^ Institute of Fermentation Sciences (IFeS), Faculty of Food and Agricultural Sciences, Fukushima University, Fukushima, Japan; ^7^ International Research and Development Center for Mucosal Vaccines, The Institute of Medical Science, The University of Tokyo (IMSUT), Tokyo, Japan

**Keywords:** Spi-B, food allergy, epithelial barrier, IgE, oral tolerance

## Abstract

Food allergy is a type I allergic reaction induced by mast cells and is mainly activated by allergen-specific immunoglobulin (Ig)E. Spi-B is an E26-transformation-specific (Ets) family transcription factor essential for the differentiation and functional maturation of several immune cell subsets, including mast cells. However, the possible involvement of Spi-B in food allergy remains unclear. In this study, we found that Spi-B-deficient mice were highly susceptible to food allergy to ovalbumin (OVA), as indicated by the exacerbation of diarrhea and elevation of serum IgE levels. These pathological changes were associated with enhanced mast cell infiltration into the intestinal lamina propria. Activation of mast cells in the intestinal mucosa was observed in *Spib*^−/−^ mice, even under physiological conditions. Accordingly, Spi-B deficiency increased the translocation of fluorescently labeled dextran from the lumen to the serum, suggesting increased intestinal permeability in *Spib*^−/−^ mice. Moreover, *Spib*^−/−^ mice showed defects in oral tolerance induction to OVA. These data illustrate that Spi-B suppresses the development of food allergies by controlling the activation of intestinal mast cells and by inducing immune tolerance to food allergens.

## Introduction

In food allergy, certain food ingredients trigger an abnormal immune response, including the induction of an antigen-specific T-helper-2 (Th2) response and impaired immune tolerance (1, 2). Food allergy symptoms can be systemic, including diarrhea, vomiting, skin rashes, and occasionally, shortness of breath (2). Immunoglobulin (Ig) E response mainly contributes to the pathogenesis of food allergies in sensitization and effector phases (3). During the sensitization phase, intimal exposure to a food allergen induces allergen-specific IgE, which binds to the high-affinity IgE receptor, Fc*ε*RI, on the surface of mast cells. Subsequent exposure to the same food allergen cross-links allergen-specific IgE to mast cells, eliciting an effector phase response. At the early stage of the effector phase, mast cells release chemical mediators, such as histamine, prostaglandins, and leukotrienes, to induce allergic symptoms. Furthermore, chemical mediators and cytokines (i.e., IL-4, IL-5, IL-13, and IL-33) produced by mast cells and basophils activate eosinophils and Th2 cells, which further amplify allergic reactions (4). Mast cell-deficient mice and mice with decreased mast cell activation show attenuated symptoms in food allergy models (5, 6), indicating that mast cells play a central role in food allergies.

The intestinal barrier system is well-developed to prevent the invasion of undesired antigens and harmful organisms. Intestinal epithelial cells form a physical barrier by tightly binding to each other through tight junctions. The thick mucus layers covering the intestinal epithelium act as physical barriers. Furthermore, secreted IgA functions as a humoral defense system to provide a first-line defense against microbes and allergens by inhibiting their attachment to the mucosal surfaces (7). Growing evidence suggests that intestinal barrier dysfunction contributes to food hypersensitivity through increased sensitization. For example, abnormal intestinal permeability has been observed in infants with food allergies (8). Furthermore, intestinal IL-9 overexpression predisposes oral antigen sensitization by increasing intestinal permeability through mast cell activation, eventually inducing intestinal anaphylaxis (9).

Spi-B is an E26-transformation-specific (Ets) family transcription factor expressed in various cells, including hematopoietic cells, plasmacytoid DCs (pDCs), microfold (M) cells, and tuft cells (10–14). This transcription factor has various functions, including the generation of lymphoid progenitor cells, B-cell proliferation and survival, and the development and functional maturation of pDCs (11, 13). Spi-B suppresses the differentiation of myeloid progenitor cells into mast cells by controlling the expression balance between PU.1 and GATA1 (15–17). Consequently, Spi-B deficiency enhances the number of mast cells. Interestingly, *Spib*^−/−^ phenotype in mice was pronounced in the intestinal tract, as manifested by the prevention of helminthic infections in the intestinal tract by activated mast cells (18). We previously reported that Spi-B is critical for the differentiation of M cells that contribute to antigen-specific IgA response by transporting luminal antigens to mucosa-associated lymphoid tissue such as Peyer's patches (PPs) (12, 19). Therefore, Spi-B contributes to maintaining immune homeostasis, especially in the intestine; however, its influence on food allergies remains unknown.

In this study, we induced food allergies in *Spib*^−/−^ mice to elucidate the association between Spi-B and food allergies. Spi-B deficiency caused severe allergic diarrhea and elevated antigen-specific IgE production. Mast cells were constitutively activated, and epithelial permeability was increased in *Spib*^−/−^ mice. Furthermore, the delayed-type hypersensitivity (DTH) response was not sufficiently suppressed by the oral administration of antigens, suggesting that *Spib*^−/−^ mice fail to develop oral tolerance. Our data illustrate the protective role of Spi-B in food allergies by securing intestinal barrier function and oral tolerance.

## Materials and methods

### Animals

BALB/cA mice were obtained from Japan Clea Co. (Tokyo, Japan). *Spib*^−/−^ mice on a C57BL/6J background were backcrossed with BALB/cA mice at least eight times. All experiments were performed on age- and sex-matched mice (5–6 weeks old) and approved by the Keio University Animal Care Committee. The mice were housed under conventional conditions and received a standard diet (AIN-93G, Oriental Yeast, Osaka, Japan) and tap water.

### Induction of food allergy

The food allergy (FA) model was created using a modified version of a previously reported method (20). Mice were intraperitoneally sensitized to 50 *μ*g ovalbumin (OVA; purity ≥98%; Sigma Aldrich, St. Louis, MO, USA) with 50 *µ*l Imject™ Alum (6 mg aluminum hydroxide and 6 mg magnesium hydroxide; Thermo Fisher Scientific, Rockford, IL, USA) once a week for a total of two times. One week after the last sensitization, the mice were orally administered OVA (1.0–1.3 mg/g body weight, purity ≥90%; Sigma Aldrich) four times every two days. Fecal consistency was visually examined within 1 h following each oral OVA administration to determine the fecal score as follows: 0, normal hard stool; 1, normal stool; 2, soft stool with shape; 3, soft stool out of shape; 4, diarrhea, liquid or without shape (21, 22).

### Intestinal permeability

Mice were fasted for 4 h and then orally administered 4-kDa fluorescein isothiocyanate (FITC)-dextran (60 mg/100 g body weight; Sigma Aldrich). After 45 min, blood samples were collected *via* the facial vein and mixed with heparin (Mochida Pharmaceutical Co., Ltd., Tokyo, Japan). The blood samples were centrifuged at 2,000 × *g* for 10 min to collect plasma, which was then serially diluted from 1:1 to 1:4 in PBS (pH 7.4; Nacalai Tesque Inc., Kyoto, Japan) and 4-kDa FITC-dextran concentration was analyzed with a fluorescence spectrophotometer (Infinite 2,000; Tecan, Männedorf, Switzerland) at an excitation wavelength of 485 nm and an emission wavelength of 535 nm. Standard curves were obtained using the values of stepwise diluted 4-kDa FITC-dextran in PBS.

### ELISA

The amounts of OVA-specific immunoglobulins in the serum and fecal samples were measured using ELISA. The 96-well ELISA plate (F96 Maxisorp Nunc-Immuno plate, Thermo Fisher Scientific) was coated with 50 *μ*l OVA/PBS (1 mg/ml) per well for 16–18 h at 4 °C. Blood samples were collected from the facial vein (days 1, 6, and 13) and the heart (day 20). After 1 h incubation at room temperature (24–26 °C), the blood samples were centrifuged at 10,000 × *g* for 3 min, and sera were collected. Sera were further centrifuged at 10,000 × *g* for 3 min to avoid contamination with blood clots. The collected sera were diluted in 1% bovine serum albumin (BSA)/PBS for ELISA analysis and the amount of OVA-specific IgE was measured using an IgE ELISA kit (#432401; BioLegend, San Diego, CA, USA) according to the manufacturer's instructions. The amount of mucosal mast cell protease-1 (MMCP-1) in serum was measured using the ELISA Kit (#88-7503-88, Invitrogen, Waltham, USA).

To measure IgA levels, fecal samples were crushed and suspended in PBS containing a protease inhibitor cocktail (cOmplete EDTA-free®; Roche Diagnostics, Risch-Rotkreuz, Switzerland). The obtained fecal suspension was centrifuged at 2,000 × *g* for 10 min to collect the supernatants. To measure IgA levels, OVA-coated ELISA plates were washed three times with 0.1% Tween-20 (Nacalai Tesque) in PBS and then blocked with 2% BSA (Nacalai Tesque) in PBS for at least 1 h. Fecal supernatants were added to the wells and incubated for 2 h. After washing, IgA targeting HRP-conjugated antibodies (1:10000 dilution; Bethyl Laboratories, Montgomery, AL, USA) were added to the plate. After 1 h incubation and four washes, 100 *μ*l TMB ELISA solution (Thermo Fisher Scientific) was added to each well and incubated in the dark. The enzymatic reaction was stopped by adding 100 *μ*l of 1.2 M H_2_SO_4_. Optical density was measured at 450 nm and 570 nm using a microplate reader (Infinite 200; Tecan).

### Flow cytometry

PPs, mesenteric lymph nodes (MLN), and spleen were isolated on day 20 (after 4th OVA challenge) and crushed using 1 ml syringe plungers and 100 *μ*m cell strainers (Greiner Bio-One, Kremsmünster, Austria). Splenocytes were treated with RBC-Lysis buffer (BioLegend) to lyse the red blood cells. After filtering with 100 *µ*m cell strainers, the single-cell suspensions were washed with HBSS (Nacalai Tesque) and resuspended with PBS containing 100 units/ml penicillin, 100 *μ*g/ml streptomycin (#26253, Nacalai Tesque), 10 mM HEPES (pH 7.3, Nacalai Tesque), and 2% newborn calf serum (NBCS, Gibco, Thermo Fisher Scientific). The samples were incubated with anti-mouse CD16/32 antibody (BioLegend) to block the Fc receptor and then stained using antibodies listed in Supplementary Table S1. To label the dead cells, 7-AAD (BioLegend) was added to the cell suspension. The cells were analyzed using a flow cytometer (BD FACSCelesta or BD LSRII; BD Biosciences, NJ, USA). The leukocyte number was determined according to the manufacturer's protocol using precision count beads (BioLegend).

### Immunostaining of mast cells

Jejunal tissues were dissected 5–6 cm from the pyloric region of the stomach. After fixation with 4% paraformaldehyde (PFA) (Nacalai Tesque) in PBS for 4 h, the tissues were placed in 30% sucrose (Nacalai Tesque) solution at 4 °C overnight. The tissues embedded in the OCT compound (Sakura Finetek, Tokyo, Japan) were quickly frozen in liquid nitrogen. Frozen blocks were sliced into 30 *µ*m thin sections. Well-dried tissue sections on glass slides were treated with 0.3% Triton X-100 (Nacalai Tesque) in PBS for 15 min and then incubated with 10% donkey serum (Sigma Aldrich) for 30 min. Next, the sections were incubated with sheep anti-MMCP-1 antibody (1 *μ*g/ml, clone #285008; R/D Systems, Inc., Minneapolis, MN, USA) and rat anti-E-cadherin (2.5 ng/ml, clone ECCD-2; Takara Bio Inc., Shiga, Japan) overnight, washed with PBS three times, and then treated with Cy3-labeled anti-sheep IgG (Jackson Immunoresearch, West Grove, PA, USA), Alexa Fluor® 488-labeled anti-rat IgG (Jackson Immunoresearch) and Hoechst 33342 (Molecular Probes, OR, USA) for 2 h. The stained sections were mounted using SlowFadeGold mounting medium (Thermo Fisher Scientific). The specimens were observed using an FV3000 confocal laser scanning microscope (Olympus, Tokyo, Japan).

### Induction of oral tolerance

Oral tolerance was induced, as described in a previous study (23). Briefly, 25 mg OVA (purity >90%; Sigma Aldrich) in 0.25 ml PBS (Nacalai Tesque) or vehicle as a negative reference were orally administered to mice. The mice were subcutaneously immunized with 100 *μ*g OVA and 100 *μ*g complete Freund's adjuvant (CFA; Becton Dickinson, and Company Franklin Lakes, NJ, USA) 7 days after oral administration. After 14 days, serum OVA-specific IgG levels were analyzed. The DTH response was examined 7 days after subcutaneous sensitization. Ten micrograms OVA in 20 *μ*l PBS or only PBS as a negative reference was administered to the right or left ear pinna, respectively. Ear swelling was measured using a micrometer (Mitutoyo, Kawasaki, Japan) 0 and 24 h after intradermal OVA administration. The DTH response was determined as the difference in thickness between the right and left ears in each mouse.

### Statistical analysis

Values are expressed as mean ± standard error of the mean (SEM). Differences between the two groups were analyzed using an unpaired *t*-test. When variances were not homogeneous, data were analyzed using the Mann–Whitney U test. Differences between more than three groups were analyzed using the one-way analysis of variance (ANOVA) followed by Tukey's test. Non-parametric data were analyzed using the Kruskal–Wallis test, followed by Dunn's test. IgE and IgA levels and DTH response data were analyzed using two-way ANOVA, followed by Tukey's test. GraphPad Prism version 9 was used for all statistical analyses. Differences were considered statistically significant at *p* values less than 0.05.

## Results

### Spi-B deficiency exacerbates the symptoms of food allergy

**Figure 1:**
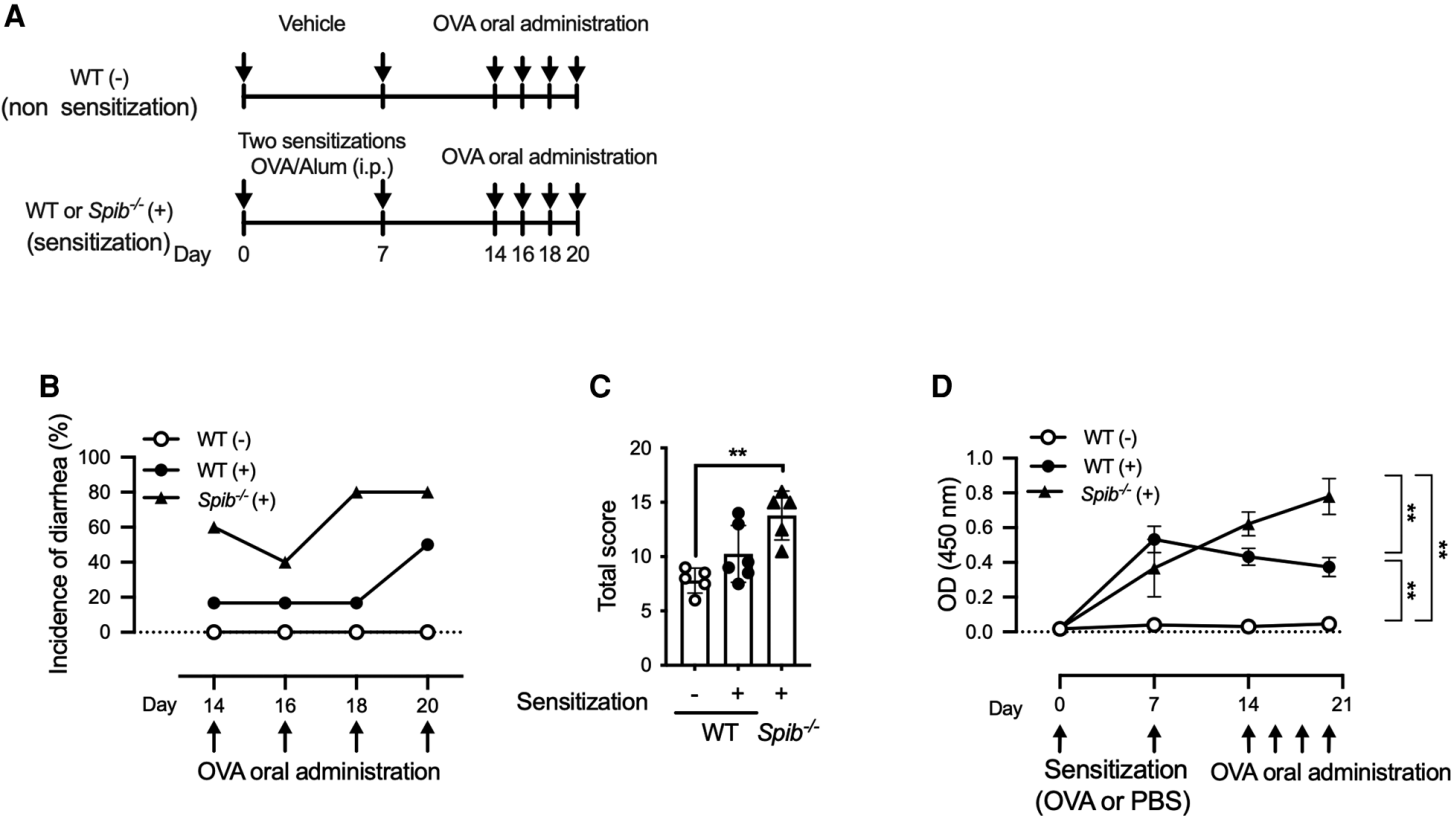
Spi-B deficiency exacerbates the symptoms of OVA-induced food allergy. (**A**) Experimental design for sensitization and oral administration of OVA to develop a food allergy model. (**B,C**) Fecal consistency was visually examined within 1 h of each oral OVA administration to determine the fecal score. The incidence of diarrhea was indicated by the fecal score of 4 (**B**) and total fecal scores during Day 14–20 (**C**). (**D**) Time course of OVA-specific IgE production after OVA sensitization. Serum samples were collected on the indicated days to measure OVA-specific IgE levels. Representative data from at least three independent experiments are shown. Data are expressed as the mean ± SEM (*n* = 5 or 6). (**C,D**) **p < 0.01, calculated using the Kruskal–Wallis test and two-way ANOVA.

We evaluated the effect of Spi-B deficiency on the development of food allergies. Since BALB/c mice are more susceptible to allergic diseases than C57BL/6 mice, we generated *Spib*^−/−^ mice with a BALB/cA genetic background. *Spib*^−/−^ and wild-type (WT) BALB/cA mice were sensitized by intraperitoneal injection with OVA and alum twice and challenged with OVA orally (Figure 1A). Fecal scores were measured as described in the Materials and Methods section. In WT mice, the incidence of diarrhea was 20% until the third challenge (day 18) and then increased up to 50% at the fourth challenge (day 20). Meanwhile, 60% of the *Spib*^−/−^ mice developed diarrhea during the first challenge, with an incidence of 80% at the third challenge (Figure 1B). Likewise, the total stool scores for the four OVA challenges tended to increase in *Spib*^−/−^ mice compared with sensitized WT control mice (Figure 1C). There was no significant difference between the stool scores of the non-sensitized *Spib*^−/−^ and WT mice (Supplementary Figure S1). These observations imply that Spi-B deficiency aggravates the development of food allergies.

### Serum OVA-specific IgE increases in *Spib*^−/−^ mice

IgE is a significant mediator of food allergic reactions. The serum levels of OVA-specific IgE in WT mice culminated on day 7 and slightly declined until day 20 (Figure 1D). In contrast, *Spib*^−/−^ mice exhibited increased serum OVA-specific IgE levels after sensitization, which further elevated until day 20 (Figure 1D). Therefore, the serum levels of OVA-specific IgE on day 20 were significantly higher in *Spib*^−/−^ mice than in WT mice (Figure 1D). Notably, OVA-specific IgE was undetectable in the serum of WT mice who were not sensitized (Figure 1D).

**Figure 2:**
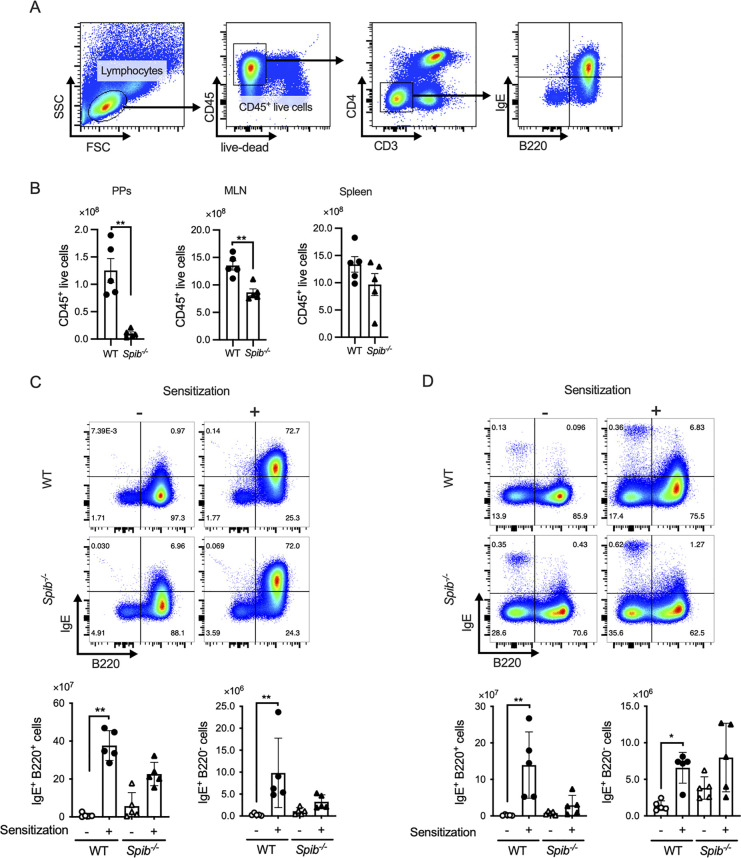
Splenic IgE^+^ cells are responsible for IgE production in *Spib*^−/−^ mice. (**A**) Gating strategy of CD3^−^ CD4^−^ CD45^+^ live lymphocytes. (**B**) The numbers of CD45^+^ live lymphocytes of Peyer's patches (PPs), the mesenteric lymph node (MLN), and the spleen from OVA-sensitized mice (day 20). (**C,D**) The CD3^−^ CD4^−^ 7-AAD^−^ CD45^+^ cells were further divided into IgE^+^ B220^−^ and IgE^+^ B220^−^ cells. The bar graphs show the number of IgE^+^ B220^−^ and IgE^+^ B220^−^ cells in the MLN (**C**) and the spleen (**D**). Representative data from at least three independent experiments are shown. Data are expressed as mean ± SEM (*n* = 5 or 6). *p < 0.05, **p < 0.01, calculated by unpaired *t*-test for B and Kruskal–Wallis test for **C,D**.

We performed flow cytometric analysis of lymphocytes from PPs, MLN, and the spleen to determine the number of IgE-producing B cells. Spi-B deficiency severely affected the total number of CD45^+^ leukocytes in the PPs of BALB/c background mice (Figures 2A,B), consistent with our previous report using *Spib*^−/−^ C57BL/6J mice (19). Therefore, we mainly analyzed the MLN and spleen in subsequent analyses. In the spleen and MLN of WT mice, OVA sensitization increased the number of IgE^+^B220^+^ and IgE^+^B220^−^ cells (Figures 2C,D). The numbers of both populations tended to decrease in *Spib*^−/−^ mice, most likely because of the reduction in total lymphocytes (Figure 2B). In contrast, the number of IgE^+^B220^−^ cells in the spleen was slightly increased in *Spib*^−/−^ mice under both non-sensitized and sensitized conditions. However, the differences were not significant between the two groups (Figure 2D). These observations suggest that the spleen may be a significant site for OVA-specific IgE response in *Spib*^−/−^ mice.

### Spi-B deficiency activates mast cells in the intestinal mucosa

**Figure 3:**
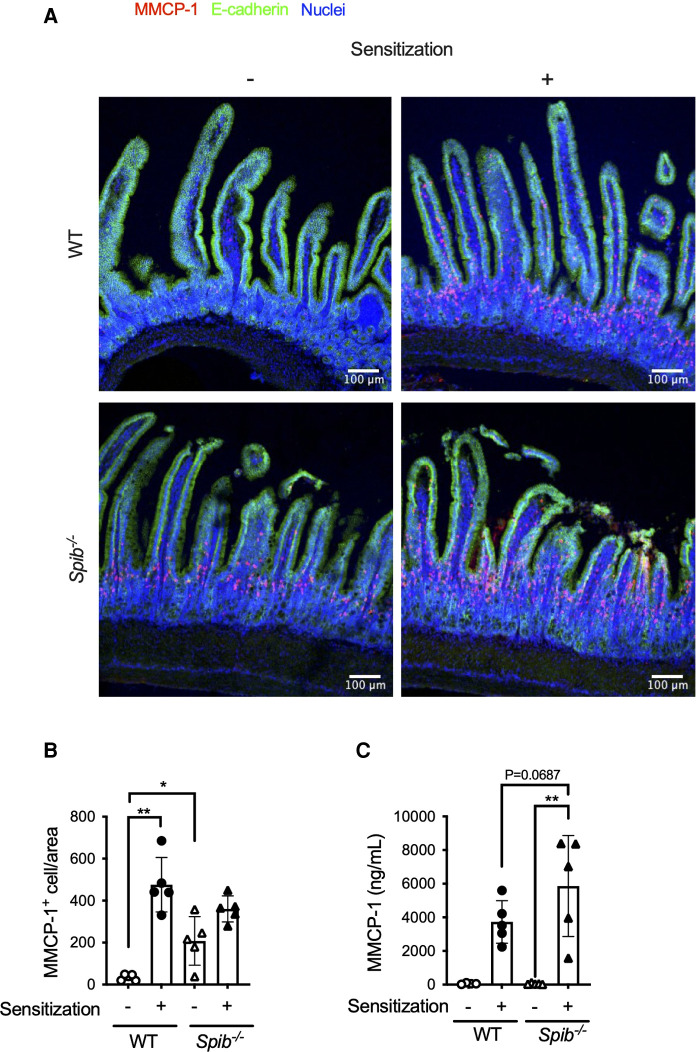
Spi-B deficiency increases intestinal mast cell activity. (**A**) Immunofluorescent images of the upper small intestine after OVA challenge (day 20). MMCP-1^+^ mast cells (red), E-cadherin (green), and nucleus (blue) are shown. Scale bars, 100 *µ*m. (**B,C**) The number of MMCP-1^+^ mast cells in the intestine (**B**) and MMCP-1 level in the serum (**C**) were measured on day 20. Representative data from at least two independent experiments are shown. Data are expressed as mean ± SEM (*n* = 5 or 6). *p < 0.05, **p < 0.01, calculated by Kruskal–Wallis test for (**B,C**).

Mast cells are the major effectors in the development of IgE-dependent food allergy symptoms. Therefore, we analyzed the number and activation status of mast cells in the intestine after oral OVA challenge, with or without sensitization. MMCP-1 is a protease primarily expressed by mast cells in mucosal tissues. Immunofluorescence analysis of MMCP-1 demonstrated that OVA sensitization facilitated the accumulation of MMCP-1^+^ mast cells in the small intestinal lamina propria (siLP) of sensitized but not non-sensitized WT mice (Figure 3A). In contrast to non-sensitized WT mice, a substantial number of MMCP-1^+^ cells was observed in the siLP of *Spib*^−/−^ mice, even under non-sensitized conditions (Figure 3A). Quantitative image analysis confirmed that the number of MMCP-1^+^ cells was significantly higher in *Spib*^−/−^ mice than in WT mice under non-sensitized conditions (Figure 3B). MMCP-1 is stored in a granular form under a steady state and is rapidly secreted by mast cells upon their activation. Indeed, the serum level of MMCP-1 was significantly elevated after oral challenge with OVA in sensitized mice (Figure 3C). Serum MMCP-1 levels tended to be higher in *Spib*^−/−^ mice than in WT mice. These results suggest that Spi-B deficiency accelerates the accumulation and activation of mast cells in the intestinal mucosa under allergic conditions (Figure 3C).

### Spi-B deficiency impaired intestinal barrier functions

**Figure 4:**
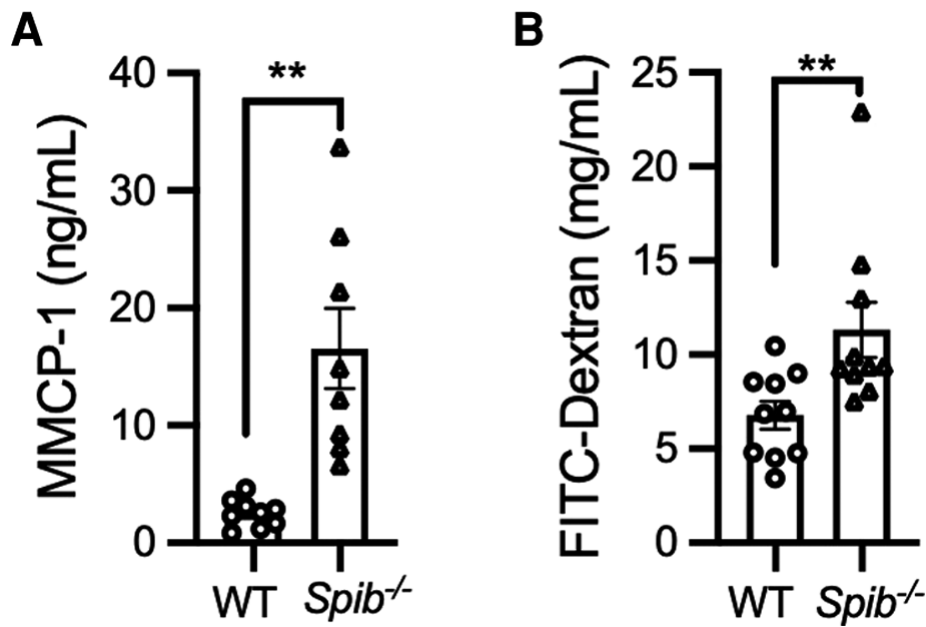
Degranulation of intestinal mast cells and increased intestinal permeability in *Spib*^−/−^ mice at the steady state. (**A**) The steady-state serum level of MMCP-1 was measured by ELISA. (**B**) The fluorescent intensity of FITC-dextran was measured in the plasma at steady-state. Representative data from at least two independent experiments are shown. Data are expressed as mean ± SEM (*n* = 10 or 11). **p < 0.01, calculated by Mann–Whitney U test.

Given that mast cells infiltrated the siLP of non-sensitized *Spib*^−/−^ mice (Figures 3A,B), we postulated that the absence of Spi-B may activate mast cells, even under physiological conditions. This hypothesis was supported by higher serum MMCP-1 levels in *Spib*^−/−^ mice than in WT mice (Figure 4A). Since activated mast cells increase intestinal epithelial permeability (7, 24), we assessed this by measuring the translocation of FITC-labeled dextran from the intestinal lumen to the blood circulation. *Spib*^−/−^ mice showed a significant increase in the amount of FITC-labeled dextran in the plasma, indicating increased intestinal permeability caused by Spi-B deficiency (Figure 4B).

IgA secretion into the intestinal mucosa establishes a humoral immune barrier to prevent penetration of external antigens, including allergens, into the body. We measured fecal OVA-specific IgA levels at the same point as the measurement of serum IgE levels (Supplementary Figure S2). OVA-IgA was detected only after the fourth oral administration (day 20) and was markedly decreased in *Spib*^−/−^ mice compared to WT mice.

### Oral tolerance is disrupted in *Spib*^−/−^ mice

**Figure 5:**
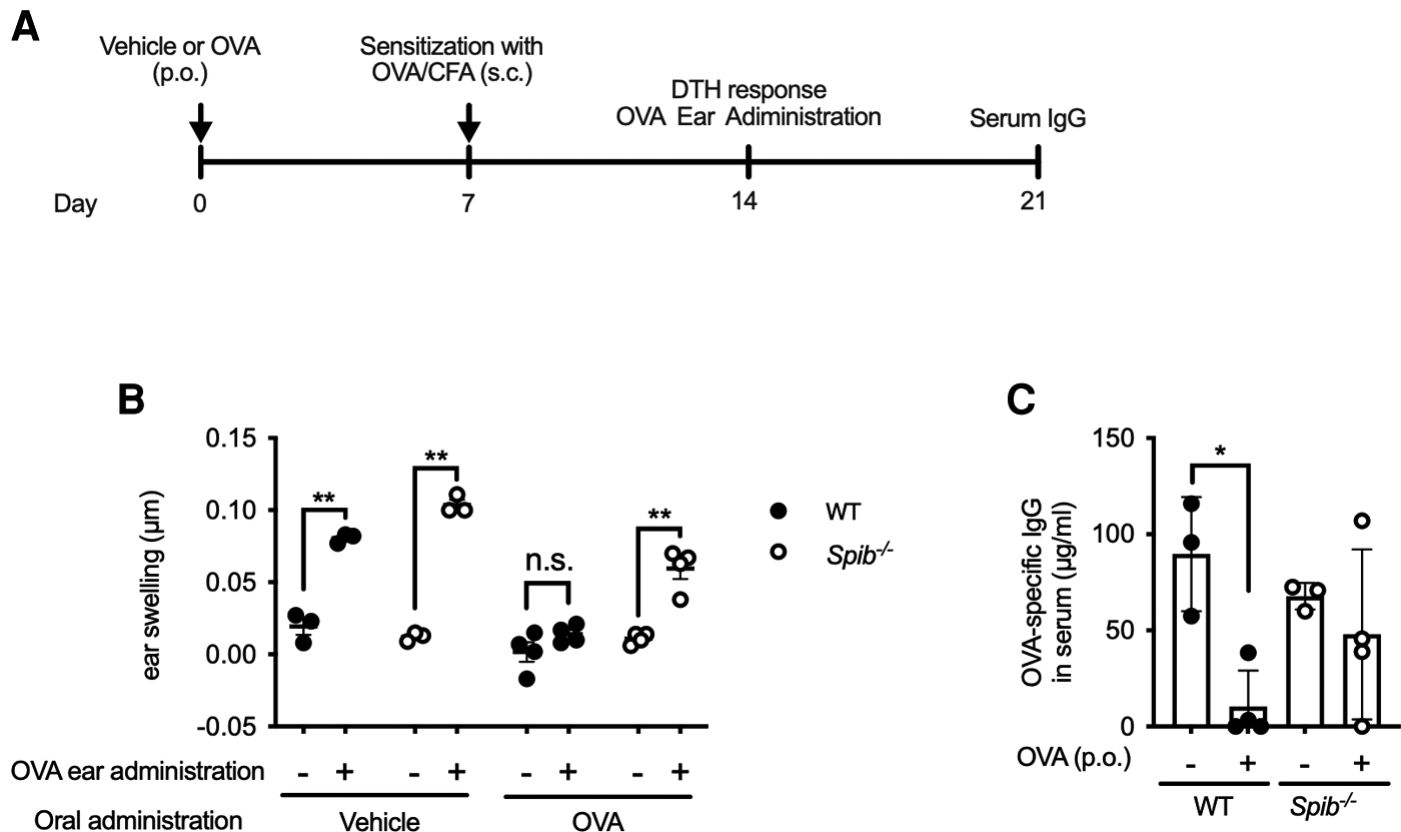
The oral tolerance is disrupted in *Spib*^−/−^ mice. (**A**) The experimental design of investigating oral tolerance. (**B**) OVA/PBS or PBS were injected in the right or left ear, respectively. The ear swelling was measured to estimate DTH response. (**C**) OVA-specific IgG levels in the serum were measured by ELISA. Representative data from at least two independent experiments are shown. Data are expressed as mean ± SEM (*n* = 3 or 4). *p < 0.05, **p < 0.01, n.s.: not significant, calculated by two-way ANOVA for (**B**) and one-way ANOVA for (**C**).

Since the failure of oral tolerance predisposes individuals to food allergies, we investigated whether Spi-B deficiency affects oral tolerance. We first examined DTH response by measuring ear swelling on day 14 (Figure 5A). The DTH response to intradermal challenge with OVA was completely suppressed in OVA-fed WT mice but not in OVA-fed *Spib*^−/−^ mice (Figure 5B).

Similarly, the induction of OVA-specific IgG was almost entirely suppressed in OVA-fed WT mice. In contrast, there was no significant difference in OVA-specific IgG levels between vehicle- and OVA-fed *Spib*^−/−^ mice (Figure 5C). Therefore, we reasoned that Spi-B was essential for oral tolerance induction.

## Discussion

The current study showed that Spi-B is a crucial regulator of food allergies in mice. Spi-B deficiency increases antigen-specific IgE levels and activates mast cells in the intestine. Consequently, the symptoms of diarrhea were exacerbated in *Spib*^−/−^ mice. Interestingly, in *Spib*^−/−^ mice, mast cells were activated even in the steady state and infiltrated into the siLP after oral administration of OVA without sensitization. Thus, mast cells were readily activated in the absence of Spi-B. Therefore, it is conceivable that excessive activation of mast cells contributes to the exacerbation of diarrhea symptoms in *Spib*^−/−^ mice. An earlier study revealed that Spi-B regulates granulocyte differentiation by balancing GATA-1 and PU.1 that directly interact with and suppress each other (15–17). Hence, Spi-B deficiency expands the mast cell population to eliminate helminth infection by promoting the activation of ILC2 (18).

We also detected increased intestinal permeability in *Spib*^−/−^ mice, which indicates an attenuated epithelial barrier. MMCP-1 release upon mast cell degranulation increases intestinal permeability in collaboration with other mast cell proteases, rMCP-II and MCPT-4, leading to allergic intestinal hypersensitivity (9, 24, 25). A recent study showed that Spi-B is essential for the proliferation of Tuft-2 cells, which sense bacterial metabolites to promote mucus secretion by goblet cells (14). Consistently, Spi-B deficiency causes thinning of the mucus layer by reducing mucus secretion from the goblet cells. Thus, the attenuated epithelial barrier in *Spib*^−/−^ mice may have resulted from the activation of mast cells and the reduction of mucus secretion. Eventually, Spi-B deficiency may enhance the translocation of food antigens from the lumen to the intestinal tissue, leading to the early onset of diarrhea (Figure 1B). However, further investigations are necessary to verify this pathological model.

Spi-B deficiency causes the loss of functional M cells (12). Because M cells are responsible for luminal antigen uptake in mucosa-associated lymphoid tissue, Spi-B deficiency severely affects intestinal microbiota-specific IgA production and cellular immunity, such as the Th17 response (19). Consistent with the results of a previous study, we observed a reduction in OVA-specific IgA production in *Spib*^−/−^ mice (19). Given that IgA suppresses attachment and invasion of antigens to the intestinal epithelium, the diminished IgA response in *Spib*^−/−^ mice may further attenuate mucosal barrier function. Nevertheless, the diminished IgA response is unlikely to contribute to the exacerbation of food allergy symptoms in *Spib*^−/−^ mice because OVA-specific fecal IgA was not detected until the late stage of food allergy (day 20) in WT and *Spib*^−/−^ mice. In contrast, the exacerbation of diarrhea was already observed at the first OVA challenge (day 14) in *Spib*^−/−^ mice.

In contrast to fecal IgA, serum levels of OVA-specific IgE were higher in *Spib*^−/−^ mice than in wild-type mice. The number of IgE^+^B220^−^ cells in the spleen under allergic conditions was sufficiently high and similar to that in the WT. The number of IgE^+^B220^+^ cells in the spleen was lower in *Spib*^−/−^ mice than in WT mice. These observations raise the possibility that Spi-B deficiency may accelerate the differentiation of IgE^+^ B cells into plasmablasts/plasma cells in the spleen, elevating the serum IgE level in *Spib*^−/−^ mice. Notably, the IgE^+^B220^+^ and IgE^+^B220^−^ cell populations in the MLN were comparable between the two groups or slightly decreased in *Spib*^−/−^ mice. While Spi-B is required for the antigen-specific IgA response and accumulation of immune cells in gut-associated lymphoid tissues, it may contribute to mitigating antigen-specific IgE responses in the systemic immune system.

Furthermore, OVA-specific IgE production in *Spib*^−/−^ mice increased continuously throughout the oral challenge, although IgE production in WT mice tended to decrease slightly during repeated oral administration of OVA. This anomaly may be attributed to the impaired oral tolerance in *Spib*^−/−^ mice. In support of this view, oral OVA administration to *Spib*^−/−^ mice failed to suppress DTH and antigen-specific IgG responses. Compromised oral tolerance is a predisposing factor for food allergies (26, 27). Therefore, exacerbation of allergic symptoms in *Spib*^−/−^ mice may at least partially result from impaired immune tolerance. Further studies are required to elucidate the mechanism by which Spi-B ensures oral tolerance. Notably, the locus of human *SPIB* is associated with primary biliary cirrhosis, an autoimmune disease (28). Thus, Spi-B may also play a role in the suppression of allergies and autoimmunity.

As the limitation study, we here employed systemic Spi-B KO mice. Because Spi-B is expressed in several types of cell lineages (e.g., myeloid progenitors, M cells, and pDCs), we were unable to identify the cell type responsible for exacerbating allergy. The generation of conditional knockout mice will elucidate the mechanisms of allergy exacerbation by Spi-B deficiency. Additionally, further investigations will be necessary to verify the pathological model that the elevation of mast cell-derived MMCP-1 due to Spi-B deficiency causes epithelial barrier dysfunction.

Within the limitation of this study, our study demonstrated the importance of Spi-B in ameliorating food allergies. Spi-B alleviates mast cell activation in the intestinal mucosa and secures intestinal barrier function and oral tolerance. Further analyses of the mucosal barrier and immunity mediated by Spi-B will improve our understanding of how immune homeostasis is maintained in the intestinal mucosa.

## Data Availability

The original contributions presented in the study are included in the article/**Supplementary Materials**, further inquiries can be directed to the corresponding author/s.
