# Supplementary material for: Spi-B alleviates food allergy by securing mucosal barrier and immune tolerance in the intestine
Source: Front Allergy. 2022 Oct 6;3:996657. doi: 10.3389/falgy.2022.996657 (PMC9584830; doi:10.3389/falgy.2022.996657)
Supplement: Supplementary file 1 [file DataSheet1.pdf]

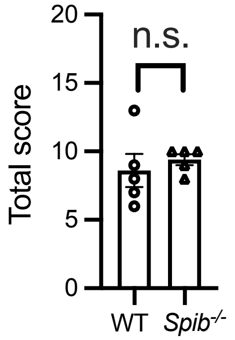

Supplemental Figure 1
Total stool score of non-sensitized WT and *Spib*$^{-/-}$ mice is shown. Fecal consistency was visually examined within 1 hour following each oral OVA administration to determine the fecal score same as described for sensitized mice. Representative data from at least two independent experiments are shown. Data are expressed as mean ± SEM (n=5). n.s.: not significant, calculated by an unpaired t test.

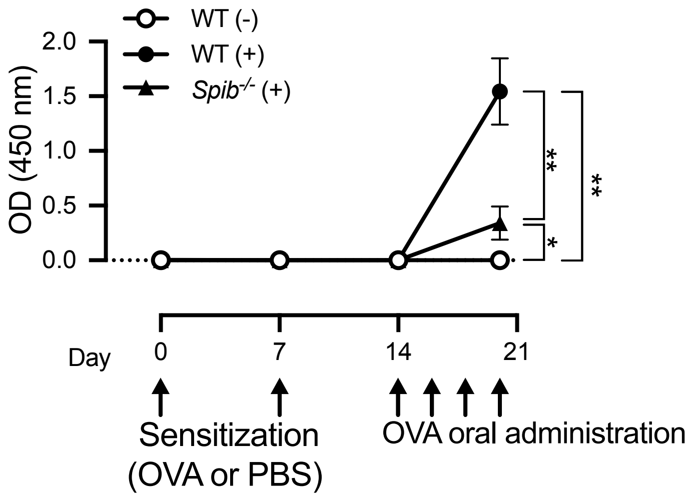

Supplemental Figure 2
The time course of OVA-specific IgA after OVA sensitization. The fecal samples were collected on the indicated day to measure the amounts of OVA-specific IgA. Representative data from at least two independent experiments are shown. Data are expressed as mean ± SEM (n=5 or 6). *P<0.05, **P<0.01, calculated by two-way ANOVA.
